# Impact of COVID-19 on Head and Neck Cancer Advancement Measured by Increasing Numbers of Urgent Dyspnea Cases—What Could Be Improved in the Event of Subsequent Pandemics?

**DOI:** 10.3390/jcm11216385

**Published:** 2022-10-28

**Authors:** Wioletta Pietruszewska, Paweł Burduk, Oskar Rosiak, Paulina Podlawska, Bartosz Zakrzewski, Magda Barańska, Magdalena Kowalczyk, Jakub Piątkowski, Grzegorz Śmigielski, Paweł Solarz, Marta Staszak, Małgorzata Wierzbicka, Bogusław Mikaszewski

**Affiliations:** 1 Department of Otolaryngology, Head and Neck Oncology, Medical University of Lodz, 22 Kopcińskiego Str., 90-153 Lodz, Poland; 2 Department of Otolaryngology, Phoniatrics and Audiology, Collegium Medicum in Bydgoszcz, Nicolaus Copernicus University, 75 Ujejskiego, 85-168 Bydgoszcz, Poland; 3 Balance Disorder Unit, Department of Otolaryngology, Medical University of Lodz, 22 Kopcińskiego Str., 90-153 Lodz, Poland; 4 Department of Otolaryngology and Laryngological Oncology, University of Medical Sciences, 49 Przybyszewskiego Str., 60-357 Poznań, Poland; 5 Department of Otolaryngology, Faculty of Medicine, Medical University of Gdańsk, 17 Smoluchowskiego, 80-214 Gdańsk, Poland; 6 Institute of Human Genetics, Polish Academy of Sciences, Strzeszynska 32, 60-479 Poznan, Poland

**Keywords:** COVID-19, dyspnea, head and neck cancer, telehealth, urgent tracheotomy

## Abstract

The COVID-19 pandemic has altered all aspects of the healthcare system’s organization and impacted patients with head and neck cancer (HNC) who have experienced delayed diagnosis and treatment. The pandemic resulted in the admission of patients with severe dyspnea and a need for tracheotomy due to extremely advanced HNC. This study’s objective was to evaluate the clinical characteristics of two multi-center cohorts, “pre-COVID-19” and “COVID-19”, of HNC patients admitted as emergencies for dyspnea. The therapeutic activity of HNC patients in four University Departments of Otolaryngology was studied over two time periods: September–February 2019/2020 and 2020/2021. A group of 136 HNC patients who underwent a tracheotomy in two-time cohorts, pre-COVID-19 (N = 59) and COVID-19 (N = 77), was analyzed. The mean tracheotomies incidence proportion was 1.82 (SD: 1.12) for the pre-COVID-19 and 3.79 (SD: 2.76) for COVID-19 period. A rise in the occurrence of emergency dyspnea was observed in the COVID-19 cohort, and the greatest increase was seen in the centers with the highest limitations on planned surgeries. In the pre-COVID-19 period, 66% of patients presented with symptoms for more than a month in comparison to 78% of patients in the COVID-19 period (*p* = 0.04). There was a higher incidence of laryngeal and laryngopharyngeal cancer in the COVID-19 period (63% vs. 75%, respectively). The number of tracheotomies performed under general anesthesia dropped in favor of local anesthesia during COVID-19 (64% vs. 56%, respectively) due to extremely advanced HNC. In the COVID-19 cohort, most patients received a telemedicine consultation (N = 55, 71%) in comparison to the pre-COVID-19 period (N = 14, 24%). Reorganization of the referral system, adjustment of treatment capacity for an increased number of HNC, and a reserve for more extensive resection and reconstruction surgeries should be made in the profile of otorhinolaryngological departments, ensuring future HNC treatment is not hampered in case of a new pandemic wave.

## 1. Introduction

The COVID-19 pandemic has changed all aspects of the organization of the healthcare system. There has been a huge reduction in the availability of medical services in certain areas of healthcare, especially in primary healthcare and outpatient specialist care [1,2,3]. A shift from specialist care to telemedicine visits was observed [4]. These changes affected head and neck cancer (HNC) patients whose diagnosis of cancer and the implementation of treatment were delayed due to telemedical visits, a limitation of inpatient services, or individual fear of contact with healthcare providers. Timeliness of care delivery is regarded as a major quality metric of oncologic care [5,6,7]. The aggressive nature of the majority of HNC negates the ability to defer surgical treatment for a prolonged period [8,9]. The consequence of this was (and still is) the admission of patients with extremely high-stage oropharyngeal and laryngeal cancers, with severe dyspnea, urgently requiring a tracheotomy.

Despite the fact that cancer patients represented a vulnerable cohort during the SARS-CoV-2 pandemic, the likelihood of SARS-CoV-2-related death was lower than the risk arising from advanced disease; however, a severe course of infection with SARS-CoV-2 had the potential to lead to the premature death of these patients [10,11]. Thus, oncology teams around the world adapted their practice with the aim of protecting patients [12]. Oncological societies created a plethora of recommendations, but precise instructions regarding routine oncological procedures remain absent [8,10,13,14,15]. A point-based scoring algorithm was developed, the Surgical Prioritization and Ranking Tool and Navigation Aid for Head and Neck Cancer (SPARTAN-HN), which consistently stratifies patients requiring HNC surgical care in the COVID-19 era [16]. Additionally, an attempt was made to rapidly implement a remote triaging system for the assessment of HNC referrals, utilizing the Head and Neck Cancer Risk Calculator version 2 [17].

However, the real problem is not with patient segregation but with their delayed and late reporting. The dominant obstruction was getting patients to the doctor in the first place. The aforementioned phenomena have been observed in clinical practice since the autumn of 2020, while now, i.e., in the spring of 2021 and on the verge of another wave of the pandemic, detailed analyses and remedial measures are required. The premise for this analysis was how to improve diagnostics, the admission system, and treatment implementation in the age of a pandemic so that patients with extremely advanced cancer seek treatment.

The aim of the study was to compare the clinical features of two multi-center cohorts of HNC patients, “pre-COVID-19” (2019/2020) and “COVID-19” (2020/2021), admitted as emergencies for dyspnea to restore the airway and to analyze the cause of the presentation delay.

## 2. Materials and Methods

The therapeutic activity in HNC patients in four University Departments of Otolaryngology (Poznań, Łódź, Gdańsk, Bydgoszcz) was compared between two time periods: September–February 2019/2020 and 2020/2021. The analyzed period was selected in accordance with the duration of the second wave of the pandemic (2020/2021), and the reference period from 2019/2020 was adjusted to it. All four departments provided services within the normal scope and with all precautionary measures. There were slight organizational differences between the units related to staff fluctuation and periodic lockdowns. Nevertheless, the differences were not the aim of the analysis, and the materials were considered jointly due to the department profile similarities.

Urgent dyspnea was defined as a significant deterioration of respiratory comfort, with inspiratory–expiratory dyspnea and a decrease in oxygen saturation below 90. The time from the patient’s registration in the emergency room to performing the procedure (tracheotomy) ranged from 15 to 180 min, with an average of 95 min ans a median of 60 min.

Diagnostic work-up: Laryngoscopy or transnasal videolaryngoscopy using flexible scopes was performed, and the necessary information was obtained from the patients’ documentation, family, or the ex-post evaluation of the surgery. Tumor location and extent in the oncology group and dynamic information on laryngeal mobility were assessed. CT imaging of the larynx was performed before the procedure.

Treatment: Tracheostomy, as the treatment of choice, was performed in all patients. There were two categories of these procedures: under general anesthesia, after transport to the operating room, with the participation of anesthesiologists and after intubating the patient, and under local anesthesia, without the possibility of intubation, in a state of extreme dyspnea, in the ER, or in a quickly launched operating room.

The “pre-COVID-19” and “COVID-19” patients were compared. The following variables were analyzed for both cohorts: age, gender, symptom duration, history of documented cardiovascular events, number of telemedicine consultations received, BMI, location of the primary tumor site, and the advancement of the disease based on the TNM system (2017) [18]. The differences in the presented variables for both pre-COVID-19 and COVID-19 cohorts were analyzed.

The first outcome measure was the incidence proportion of urgent dyspnea cases requiring a tracheotomy in the course of advanced head and neck neoplasms in relation to the total number of all surgical procedures in otorhinolaryngological centers in both time periods. The second outcome measure was a detailed description of the specificity of the COVID-19 group.

### Statistical Analysis

Statistical analysis was performed using STATISTICA software (Version 13.1, Dell, Austin, TX, USA). The incidence proportion for laryngeal dyspnea related to tumors of the head and neck was calculated by dividing the total number of surgical procedures related to emergency dyspnea due to neoplasm by the total number of surgical procedures in each given time in each clinical center individually, with the value presented as a percentage.

The Shapiro–Wilk test was used to assess the normal distribution of the continuous variables, which were summarized using the mean and standard deviation (SD) for normally distributed variables, and median values and interquartile ranges for non-normally distributed variables.

Comparisons between the pre-COVID and COVID cohorts for continuous and normally distributed variables within the groups were performed by first performing Levene’s test to check for equality of variance between the groups, and upon rejecting the null hypothesis in all instances, the Student’s *t*-test for independent samples was performed. For non-normally distributed continuous variables, the U-Mann–Whitney test was conducted. To analyze the effect of nominal variables between the cohorts, contingency tables were drawn, and Pearson’s Chi-squared test was utilized, while in populations smaller than 10 subjects, Fischer’s exact test was used. The level of significance used for all the analyses was 2-tailed and set at *p* < 0.05.

## 3. Results

The analysis concerns a group of 136 patients who underwent a tracheotomy in two-time cohorts, pre-COVID-19 (N = 59) and COVID-19 (N = 77). The study group included 105 men (pre-COVID-19: N = 45 vs. COVID-19: N = 60) and 31 women (pre-COVID-19: N = 14; COVID-19: N = 17). The mean age in the study group was 63.93 years (SD 10.48) (pre-COVID-19: 64.05 years (SD 8.79); COVID-19: 63.83 years (SD 11.67)). There were no statistically significant differences in the gender distribution between the periods (Chi-squared test *p* = 0.82) or between the ages of the patients (t-student test *p* = 0.99).

The mean incidence proportion for all centers calculated for the pre-COVID-19 period of 2019/2020 was 1.82 (SD: 1.12) for the pre-COVID-19 period and 3.79 (SD: 2.76) for the COVID-19 period, respectively. A rise in the occurrence of emergency dyspnea was observed in the COVID-19 cohort, with the biggest proportional change observed in Lodz and the smallest in Poznan. There were statistically significant differences between the time cohorts (Table 1). The incidence proportion of surgical procedures due to laryngological dyspnea from head and neck cancer was summarized in Table 1.

The differences between otorhinolaryngological centers were not the aim of the analysis, and the huge number of tracheotomies in Lodz was related to it being the only emergency department in the region with a long tradition of oncology. In Gdańsk, there are another three otorhinolaryngological emergency departments with such an oncological profile.

The rise in incidence was best seen in centers which had the highest limitations due to local restrictions in planned surgeries; in Lodz, the number of planned surgeries changed significantly between the periods, causing a significant increase in the incidence of emergency procedures due to laryngological dyspnea in the course of oncologic disease, while for centers which noted less significant changes in the total number of planned surgeries (Poznan, Gdansk), the increase in tracheotomy incidence was less visible (Table 1).

All the University clinical centers noted an increase in the total number of performed tracheotomy procedures in patients with head and neck neoplasms, with the greatest increase between the pre-COVID-19 and COVID-19 periods in Gdansk by 133% and in Lodz by 33%. In Poznań and Gdańsk, there was the smallest percentage decrease in the total number of all planned surgeries performed in the COVID-19 period. In these two departments, the percentage proportion increase in tracheotomies was lower than in Łódź and Bydgoszcz. The increase in the percentage of tracheotomies as a total of surgeries performed was the most pronounced in Łódź and Bydgoszcz, but these departments were also affected by the longest period of interruption of specialist services due to the pandemic, with only urgent cases being admitted, which constituted a growing percentage of the number of total surgeries.

In Łódź and Bydgoszcz, there were similar treatment restrictions: in the COVID-19 period, these regions saw a reduction of up to 50% in the number of beds, reduced appointments numbers at the outpatient clinic, limited access to the operating room (only 2 or 3 days a week instead of 5) and an increased inflow of urgent patients from the region due to the closure of other otorhinolaryngological departments.

Epidemiological data for patients treated by urgent open tracheotomies in both pre-COVID-19 and COVID-19 cohorts are presented and compared in Table 2. The duration of symptoms was categorized to represent the urgency of the procedure into symptoms of dyspnea developing in a month or less and longer than one month. In the pre-COVID-19 period, 66% of patients presented with symptoms developing for more than a month, whereas in the COVID-19 period, as much as 78% of patients fell into this category. This observation was statistically significant (*p* = 0.04). Neither the advancement of the disease nor the localization of the primary tumor differed significantly between the groups. However, there was a higher incidence of laryngeal and laryngopharyngeal cancer in COVID-19 compared to the pre-COVID-19 period (63% vs. 75%, respectively). Other analyzed parameters, such as concomitant disorders categorized by the system (cardiovascular, respiratory, neurological, number of cardiovascular incidents, diabetes mellitus) or others, as well as concurrent neoplasms, were not found to be significantly different between the time cohorts. Obesity or malnutrition was not a significant factor since the median BMI in the pre-COVID-19 cohort was 22.5 (IQR 5.2) and in the COVID-19 cohort, 23.53 (IQR 5.54), respectively, both of which can be interpreted to be normal BMIs.

Due to the difficulties in intubating a patient with acute dyspnea limiting the ability to visualize the glottis, the number of tracheotomies performed under general anesthesia dropped in favor of those conducted under local anesthetic during COVID-19 (64% in pre-COVID19 vs. 56%, respectively).

Regarding telemedical services, which were uncommon in the pre-pandemic time, out of 59 patients, only 14 individuals (24%) received a telemedical consultation before admission to the hospital, and all those cases presented with symptoms developing for more than 30 days. In comparison, in the COVID-19 cohort, most patients received a telemedicine consultation from their primary care facility (55 patients, 71%), of which 49% of patients received at least one and 22% more than one teleconsultation. Analyzing symptom duration in this cohort of patients, only one individual with symptoms presenting for less than 7 days received a teleconsultation. Patients who presented with symptoms developing for more than 7 days, but less than 30 days, received at least one teleconsultation in 64% of all those cases.

Considering the number of teleconsultations regarding the time of developing acute dyspnea in the COVID-19 cohort in the urgent group (1–2 days of dyspnea), 21 patients (61%) received one teleconsultation and 6 (17%) more than one. In contrast, in the group which developed dyspnea for more than 3 days, 22 patients (36%) received at least one, while almost 50% of individuals received two or more teleconsultations. This observation was statistically significant (Fischer’s exact test with Freeman–Halton extension *p* = 0.036). In the pre-COVID-19 period, all individuals who received a teleconsultation (14 patients) presented with dyspnea developing over longer than 7 days.

## 4. Discussion

We presented, for the first time, a cohort analysis of the therapeutic activity of emergency otorhinolaryngological units during pandemic conditions in terms of urgent dyspnea in HNC patients. We based our observations and conclusions on a comparison of the patients admitted in the periods September–February 2019/2020 and 2020/2021. As an overview of the total medical activities of the otorhinolaryngological department, we summarized the number of services provided. Overall, a decrease in planned surgeries was observed, which ranged across different centers from −48% to −10%, dependent on the local exacerbation of the pandemic and locally imposed healthcare restrictions in normal hospital function.

Our analysis of four University Departments of Otolaryngology showed that the number of urgent tracheotomies in HNC patients increased in all of them, but proportionally, most were observed in those where the primary activity was limited. On the one hand, it proves that the system of admissions and life-saving procedures was maintained, although some of these departments were converted to meet the needs of the pandemic. However, on the other hand, the greater the involvement in combating the pandemic, the fewer planned interventions were implemented and the higher the percentage of emergency procedures. A large proportion of patients awaiting planned surgeries had them postponed, which showed that capacity was fixed, with no possibility of expanding the department’s activity. Our findings have been partially reflected in up-to-date literature. The coronavirus pandemic and the subsequent need for disease transmission mitigation efforts have significantly altered the delivery of cancer care [1,2]. The utilization of inpatient care and subsequent hospitalization deficit was estimated in developed countries to be 7–35% in oncology [19,20,21,22]. All oncological subspecialties in the US experienced significant decreases in new patient visits and surgery capacity during COVID-19 [23], with a 25% reduction in newly diagnosed head and neck malignancies [24].

In our experience, the limitations of cancer care were not reflected in the number and proportion of urgent tracheotomies in the case of cancer care between the two cohorts, though the total number did decrease for other reasons. Regardless of their place of residence, oncological patients with extreme dyspnea are mainly neglected health cases. Another observation is the change in the proportion of primary lesions and the overrepresentation of laryngeal cancer. This is probably related to the fact that during the pandemic, primaries with a rapid progression could not wait anyway, while those with slowly developed cancers, adapted to dyspnea, more often reported in an extreme state.

Our results point out that telemedicine was a popular means of providing healthcare during the pandemic, which is also observed across different healthcare systems [17,22,25]. Patients developing acute, dynamic dyspnea mostly had one telemedical consultation, which led to an immediate referral to the hospital. Full analysis of the effects of telemedicine was limited due to missing data regarding the time between the actual consultation and the patient’s date of admission to the hospital. Nonetheless, patients who presented with acute dyspnea directed to the hospital constituted a minority of cases in the COVID-19 cohort. Comparing the pre-pandemic use of telemedicine to the pandemic period, all the individuals in the COVID-19 cohort in this study, who underwent a telemedical consultation, developed symptoms over a longer period and constituted a minority of all cases in that cohort. The lack of standardized IT platforms may have resulted in serious challenges in replacing frontal visits, often making a concrete reduction of patients’ hospital access unfeasible [22]. Although telemedicine may improve healthcare access, patient preferences, technology-related barriers, and limitations regarding cancer surveillance must be addressed moving forward [25]. When given a choice, patients with head and neck cancer preferred in-person visits over telemedicine [25].

**Figure 1:**
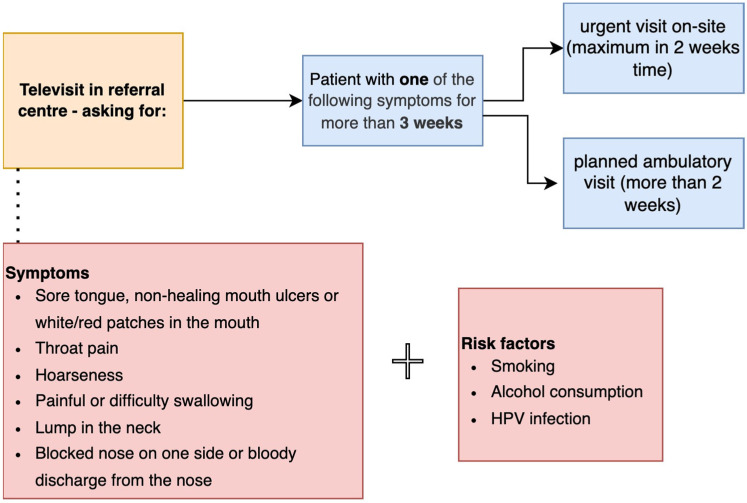
Proposed referral algorithm for patients with suspicious head and neck symptoms.

During the COVID-19 pandemic, telemedicine was implemented as a measure to manage difficulties posed by suspended ambulatory visits and the decreased number of available specialists [26,27]. However, several studies confirmed that telemedicine in otorhinolaryngology could be effectively implemented in healthcare with positive effects and patients’ experience, especially in laryngology and head and neck surgery [28,29]. Below, we provide a management scheme for patients with suspicious symptoms to maintain ongoing access to the specialist and provide an early diagnosis (Figure 1) with qualification criteria based on the white paper by the Make Sense Campaign [30]. The suggested system may be used in both patients referred by primary care doctors or follow-up patients. The central part consists of a telehealth center, in which patients are assessed in terms of alarm symptoms and divided into urgent and non-urgent groups.

Implementation of this system may lead to better compliance in follow-up patients and earlier diagnosis of head and neck malignancies.

Although the differences were not the aim of the analysis, we noticed some specificity between the four university departments. Initial surgical load in the pre-COVID-19 period, the number of emergency departments in the given regions, and breaks/closures due to the pandemic constitute some differences in the number of procedures. The density of otorhinolaryngological departments in the region and round-the-clock emergency care availability, a reduction of available bed numbers, reduced appointment numbers at the outpatient clinic, as well as limited access to the operating room also mattered.

Strengths and limitations of the study:

To our knowledge, this study constitutes the largest clinical group analyzed regarding emergency department services related to severe dyspnea in HNC patients.

One of the strongest limitations of this study was that it was a relatively small study group, which was then divided into two cohorts. Sample sizes below five individuals for some variables required the utilization of the conservative Fischer’s exact test. Inclusion of other centers might improve outcomes towards observed tendencies by expanding the analyzed population size.

In summary, the number of tracheotomy procedures in HNC patients was higher during the COVID-19 period. This may indicate both the higher advancement of a larger number of patients as well as higher reporting to the four analyzed departments, probably due to the limited availability of other healthcare providers. However, the first premise should be adopted because HNC patients are directed to the analyzed university departments anyway. However, this premise will require confirmation in subsequent analyses, i.e., the total number of patients from the oncological pathway.

The degree of advancement of HNSCC during urgent tracheotomy is the same, which is logical, but the higher percentage of tracheotomies performed under local anesthesia is noteworthy. It is a category of extremely urgent procedures with complete airway obstruction. This means that in these patients, intubation was attempted, but the glottis was not visualized, so the tracheotomy was performed immediately, or the procedure was even performed in the Emergency Department without intubation attempts.

## 5. Conclusions

In the profile of otorhinolaryngological departments, the reorganization of the referral system may improve the access of head and neck oncological patients to specialists during further COVID-19 pandemic waves. That may include direct indications for hospital telemedicine centers’ employees to the proposed algorithm, adjusting treatment capacity for an elevated number of HNC, and a reserve for more extensive resection and reconstruction surgeries.

## Figures and Tables

**Table 1:** Number of tracheotomy procedures, total number of ENT surgeries, and the incidence proportions for tracheotomy due to head and neck cancer in pre-COVID-19 and COVID-19 time cohorts. TRA—Urgent tracheotomy; SUR—Total number of ENT surgical procedures excluding tracheostomies.

		Pre-COVID-19 Cohort	COVID-19 Cohort	*p*-Value
Lodz	TRA	30	40	<0.0001
	SUR	1022	504	
Poznan	TRA	15	17	0.5160
	SUR	1505	1354	
Bydgoszcz	TRA	11	13	0.2015
	SUR	393	274	
Gdansk	TRA	3	7	0.1226 *
	SUR	416	335	
All centers	TRA	59	77	0.0010
	SUR	3336	2467	

* Calculated using Chi-squared testing unless otherwise indicated (Fisher Exact test).

**Table 2:** Patients who developed dyspnea due to advanced head and neck cancer were treated with an open surgical tracheotomy in two-time cohorts: pre-COVID-19 and COVID-19.

	Pre-COVID-19 Cohort	COVID-19 Cohort	*p*-Value
	Total N = 59	Total N = 77	
Primary tumor site			*p* = 0.34 *
Larynx and laryngopharynx	37 (63%)	58 (75%)	
Oral cavity and tongue	7 (12%)	8 (10%)	
Oropharynx	10 (17%)	6 (8%)	
Other (thyroid, esophagus, lung)	5 (9%)	5 (7%)	
Tumor advancement			*p* = 0.52 *
T2	3 (5%)	3 (4%)	
T3	13 (23%)	24 (32%)	
T4	41 (72%)	49 (65%)	
Comorbidities			
Diabetes	7 (12%)	11 (14%)	*p* = 0.80 *
Other neoplasms	6 (10%)	7 (9%)	*p* = 1.0 *
Respiratory disease	2 (3%)	6 (8%)	*p* = 0.24 *
Cardiovascular disease	22 (37%)	26 (34%)	*p* = 0.67 **
Tracheotomy under general anesthesia	38 (64%)	43 (56%)	*p* = 0.31 *
Gender			*p* = 0.82 **
Male	45 (76%)	60 (78%)	
Female	14 (24%)	17 (22%)	
Time from first symptoms to hospital admission			*p* = 0.04 *
More than 30 days	30 (66%)	60 (78%)	
8 to 30 days	10 (17%)	3 (4%)	
Less than 7 days	10 (17%)	14 (18%)	
Acute dyspnea duration			*p* = 0.65 **
1–2 days	21 (55%)	36 (62%)	
3–7 days	17 (45%)	22 (38%)	
Telemedicine consultations			*p* < 0.01 **
None	59 (100%)	22 (26%)	
One	0 (0%)	38 (49%)	
More than one	0 (0%)	17 (22%)	

* Two-tailed Fischer’s exact test. ** Pearson’s Chi-squared test with Yates’s correction for continuity.

## Data Availability

The data presented in this study are available on request from the corresponding author.
